# Genome sequence of *Escherichia coli* H1G2: a B1 lineage isolated from the gut of Hilsa shad (*Tenualosa ilisha*) of Bangladesh

**DOI:** 10.1128/mra.00138-26

**Published:** 2026-04-20

**Authors:** Fahim Al Galib, Abdullah Al Kafi, Santu Biswas, Soharth Hasnat, Dipali Rani Gupta, M. Nazmul Hoque, Md. Morshedur Rahman, Md. Mahbubur Rahman, Tofazzal Islam

**Affiliations:** 1 Institute of Biotechnology and Genetic Engineering (IBGE), Gazipur Agricultural University (GAU) 198780, Gazipur, Bangladesh; 2 Department of Gynecology, Obstetrics and Reproductive Health, Molecular Biology and Bioinformatics Laboratory, Gazipur Agricultural University 198780, Gazipur, Bangladesh; 3 Department of Dairy and Poultry Science, Gazipur Agricultural University (GAU) 198780, Gazipur, Bangladesh; University of Maryland Baltimore, Baltimore, Maryland, USA

**Keywords:** complete genome, gut microbiota, Hilsa gut, symbiotic, Oxford Nanopore sequencing

## Abstract

We characterized the 4.8 Mbp genome of *Escherichia coli* H1G2, a B1 lineage isolated from the gut of Bangladesh’s national fish, Hilsa (*Tenualosa ilisha*). The complete assembly reveals specialized genes for sugar metabolism and adhesion, facilitating niche-specific colonization, thereby providing insights into the microbial ecology of this commercially significant species' gastrointestinal tract.

## ANNOUNCEMENT

*Escherichia coli* is a vertebrate gut bacterium that supports host health (1, 2). While well-studied in humans, genomic insights into strains from aquatic hosts like Hilsa fish remain limited (3, 4). The *E. coli* was isolated from Hilsa shad (*Tenualosa ilisha*) gut captured from the Meghna River in Chandpur, Bangladesh (22.8333°N, 90.8333°E). Gut contents were aseptically removed, and a 1 g pooled sample was homogenized in 9 mL of phosphate-buffered saline (PBS, pH 7.0), enriched in MacConkey broth (37°C for 24 h at 180 rpm), and plated on eosin methylene blue (EMB) agar (Oxoid, UK) with incubation at 37°C for 24 h (3, 4). A single green metallic sheen colony was purified and designated H1G2 and was initially confirmed as *Escherichia* sp. by PCR amplification of the 16S rRNA gene (27F/1492R), followed by bidirectional Sanger sequencing (5). Genomic DNA from an overnight (37°C) EMB culture was extracted using the FavorPrep Tissue Genomic DNA Extraction HE Mini Kit (Favorgen, Taiwan) and quantified with a Qubit 4.0 fluorometer (Thermo Fisher Scientific). Libraries were prepared using the Oxford Nanopore Technologies (ONT) Ligation Sequencing Kit SQK-NBD114.24 with the NEBNext companion module (New England Biolabs) and Native Barcoding Kit 96 (v14) (no shearing and no size selection) and sequenced on an ONT MinION using an R10.4.1 flow cell (6). Basecalling with Dorado v1.2.0 (SUP model) yielded 62,359 reads totaling 391,812,180 bp (read *N*_50_, 11,906). Read quality was assessed with NanoPlot v1.46.1 (7); adapters were trimmed with Porechop v0.2.4 (8); reads >1 kb were filtered with Filtlong v0.2.1 (9); *de novo* assembly was performed with Flye v2.9.5-b1801 (10); and completeness and contamination were evaluated with BUSCO v6.0.0 (*-l bacteria_odb10, -m genome*) (11) and CheckM v1.2.4 (*lineage_wf*, *Escherichia coli* marker set) (12). The primary annotation present in the GenBank submission was generated via National Center for Biotechnology Information (NCBI) PGAP v6.10 (13). Furthermore, RAST v2.0 (14), PlasmidFinder v2.0.1 (15), and CRISPRCasFinder v2.0.3 (16) were utilized to identify specific metabolic features, plasmids, and CRISPR arrays, respectively. Manual curation was utilized to resolve differences in annotation. Default parameters were used for all software unless otherwise specified. The genome without rotating at specific base comprises a complete 4,779,014-bp circular chromosome (51% GC) and two plasmids (circular) with 4,516 protein-coding genes (Table 1). Phylogenomic analysis assigned H1G2 to *E. coli* phylogroup B1, sharing 99.24% average nucleotide identity (ANI) with the commensal strain SE11 by GTDB-Tk v2.6.1 (17); *in silico* serotyping identified O40:H2 (18); BLAST against VFDB (19) identified it lacks major diarrheagenic toxins (e.g., Shiga toxin, *east1*) and pathogenic adhesins (*aggR*, *aatA*). However, it possesses genes for adhesion (*type-1 fimbriae*, *curli*), iron acquisition, vitamin K₂ biosynthesis, and pathways for utilizing mucus-derived sugars—including fucose (*fucI*, *fucP*), galactose (*gal* and *mgl* operons), mannose (*manXYZ*), and N-acetylglucosamine (*nagE*, *nagA*), along with vitamin B₁₂ uptake and colicin V production. These genetic features suggest specialized adaptation as a potentially beneficial commensal bacterium within the Hilsa gastrointestinal tract.

**TABLE 1:** Genomic features of *E. coli* strain H1G2 isolated from the Hilsa fish gut

Genome feature	H1G2
GenBank accession	JBSVSG000000000
Assembly accession	GCA_054403135.1
SRA	SRR37373213
Number of reads	62,359 (*N*_50_ 11,906)
Assembled genome size (bp)	4,779,014 bp
GC content (%)	51
Coverage (×)	153×
Genome completeness (%)	100% (BUSCO), 99.36% (CheckM)
Contamination (%)	0.23%
BioSample accession	SAMN53736888
16S GenBank	PZ053874
*N*_50_ value (bp)	4,734,525
Number of contigs	3 (1 chromosome, plasmid)
Genes (total)	4,631
Coding sequences (CDSs)	4,516
Genes (coding)	4,384
CDSs (with protein)	4,384
Genes (RNA)	115
rRNAs	8, 7, 7 (5S, 16S, 23S)
Complete rRNAs	8, 7, 7 (5S, 16S, 23S)
tRNAs	85
ncRNAs	8
Pseudo genes	132
CDSs (without protein)	132
Pseudo genes (ambiguous residues)	0 of 132
Pseudo genes (frameshifted)	57 of 132
Pseudo genes (incomplete)	68 of 132
Pseudo genes (internal stop)	31 of 132
Pseudo genes (multiple problems)	22 of 132
Pseudo Genes (short protein)	1 of 132
CRISPR arrays	1
Plasmid replicon	2
Number of subsystems (metabolic features)	377

While metagenomic surveys previously identified *E. coli* in the Hilsa gut (20, 21), this study for the firs time reports the complete genome sequence of strain H1G2, a B1 phylogroup lineage indigenous to this host, offering critical insights for adaptation within aquatic environments.

## Data Availability

The complete genome sequence of *E. coli* strain H1G2 has been deposited in GenBank assembly under accession number GCA_054403135.1, within BioProject PRJNA1055760 (Mining Bangladesh Biogold), and Biosample SAMN53736888. The version described in this paper is JBSVSG000000000.
